# Supplementary material for: Are carbon emissions trading and green financial instruments synergistic? -Comprehensive quantitative research based on content analysis
Source: PLoS One. 2024 Mar 7;19(3):e0298601. doi: 10.1371/journal.pone.0298601 (PMC10919601; doi:10.1371/journal.pone.0298601)
Supplement: S1 Data — (ZIP) [file pone.0298601.s001.zip › Data/地级市Z_F工作报告.zip!/╜¡╦╒/─╧═¿╩╨/└┤╫╘╣1⁄2╓┌║┼í╛┬φ┐╦╩2╛▌═°í┐_2017.docx]

各位代表：

　　现在，我代表南通市第十四届人民政府向大会报告工作，请予审议，并请政协委员和其他列席人员提出意见。

　　一、本届政府工作回顾

　　市十四届人大一次会议以来，我们在以习近平同志为核心的党中央坚强领导下，深入贯彻省委、省政府和市委决策部署，主动适应经济发展新常态，自觉践行新发展理念，与时俱进推动全面小康社会建设，“迈上新台阶、建设新南通”取得重要进展。

　　推动经济稳中求进，综合实力显著增强。坚持调速不减势、量增质更优，综合施策，有效应对经济下行压力。2016年，实现地区生产总值6768亿元，比2011年增长65.5%，占全省比重从8.3%提高到8.9%，在全国大中城市排名从27位前移至21位；一般公共预算收入590亿元，五年增长58%。国家创新型试点城市、质量强市示范市和知识产权示范城市建设初见成效，全社会研发投入占地区生产总值比重从2.1%提高到2.61%，科技进步贡献率从55.5%提高到61%，高新技术企业达930家，国家“千人计划”人才145名，省“双创”人才260名。三次产业结构优化为5.4∶46.8∶47.8，服务业增加值占地区生产总值比重提高9.3个百分点，高新技术产业产值居全省第二、占规模以上工业比重提高9.3个百分点。上市公司和“新三板”挂牌企业109家，成为全国第5个存款超万亿元地级市。获批省建筑产业现代化示范城市。

　　加快优江拓海进程，开发格局实现突破。抢抓多重国家战略叠加机遇，规划引领，交通先行，推动沿江转型升级、沿海科学开发、陆海统筹发展。崇启大桥建成通车，沪通长江大桥投资完成率48%，宁启动车开通运营，兴东机场建成国际机场，新增一级以上公路669公里、内河高等级航道159公里，实现高速公路县县通和临海与沿江高等级公路环通，长江南通段12.5米深水航道通航，奠定了建设区域性综合交通枢纽坚实基础。“一港八区”江海组合大港加速形成，整治沿江沿海岸线25.3公里，围垦滩涂6340公顷，继续保持耕地占补平衡，成为国家海域综合管理创新示范市，沿海前沿区域生产总值和投资分别增长93%、162%。

　　加大改革开放力度，体制创新成效明显。突出问题导向跟进改革，在解放思想中激发活力，在改革创新中破解难题，在扩大开放中增创优势。陆海统筹发展、相对集中行政许可权、综合行政执法、市场采购贸易方式等列入国家级改革试点，陆海统筹发展综合改革6个方面体制机制创新有序推进，实施重点领域改革35项；行政审批制度改革全国领先，非许可审批事项全面取消，“一枚印章管到底”被国务院第三次大督查确定为典型经验做法并通报表扬；市级基本实现“一个部门一支队伍管执法”。海安、如皋、海门开发区和南通高新区升格为国家级，南通综合保税区获批，新增洋口港、启东港、兴东机场3个国家一类开放口岸；设立通州湾江海联动开发示范区，新增省级开发园区7个，建成跨国跨江合作园区15家。累计实际利用外资115.4亿美元，进出口总额居全省第四，外经合作保持全省领先。私营个体企业总数居全省第三，16家企业跻身全省民营企业百强。获批国家级海峡两岸文创产业合作区和青年创业基地。

　　推进城乡发展一体化，环境面貌加速提升。促进“四化”同步发展，统筹生产、生活、生态空间。五级城乡体系初步形成，常住人口城镇化率从57.6%提高到64.4%；市区组团发展格局拉开，快速畅通工程全面推进，建成区面积由140.9平方公里拓展到215.6平方公里；5个县（市）和19个市级中心镇功能加快提升，基础设施和公共服务不断向农村延伸。农业基本现代化水平居苏中苏北首位，国家现代农业示范区和农业科技园区加快建设，高效设施农（渔）业179.8万亩。绿色发展指数全省第一，中水回用破解造纸制浆废水零排放难题，林木覆盖率24%，城乡环境综合整治深入开展，建成国家生态市。

　　强化社会建设治理，人民生活持续改善。推进民生幸福“十大体系”建设，发展成果更多更公平地惠及广大群众。五年兴办重点民生实事224项，财政民生支出占比达76%；城镇、农村居民人均可支配收入分别达39247元、18741元，分别增长58.4%、65.1%；居民储蓄存款增长75.8%；年收入低于5000元人口全部脱贫，新一轮脱贫致富奔小康工程启动，完成10.89万年收入7000元以下低收入人口建档立卡。全面普及15年教育，成为全国首批义务教育发展基本均衡地级市，新增本科高校1所、高职高专2所、省联院分院4所；新增三级医院9家，乡镇卫生院示范化率70%；万人公共文化体育设施面积增长21%；每千名老人养老床位数33.9张。五大保险参保率97.5%，基本照护保险试点经验全国推广。市区动迁异地安置基本实现“先建后征”。平安南通、法治南通建设成效显著，公众安全感、法治建设满意度居全省前列，蝉联“六五”普法全国先进市。中国好人、道德模范、最美人物等精神文明先进典型持续涌现，江海志愿者队伍不断壮大，实现全国文明城市“三连冠”、全国双拥模范城“六连冠”。

　　2016年，面对严峻复杂的外部环境和困难挑战，我们按照省委、省政府和市委要求，坚持稳中求进，狠抓工作落实，推动好上又好、能快则快发展，为十四届政府工作画上了圆满句号。全市地区生产总值增长9.3%，一般公共预算收入同口径增长0.5%，城乡居民收入分别增长8.1%和8.5%，居民消费价格涨幅2.3%，城镇登记失业率1.85%，省下达节能减排任务全面完成，实现了“十三五”良好开局。这一年，着重做了以下工作：

　　（一）坚持稳增长与调结构并举。围绕项目投资总量和质量“两个再上新台阶”，猛攻有效投入，固定资产投资4812亿元、增长10%，省级、市级重大项目分别完成投资298亿元和830亿元，争取国家专项建设基金70亿元，新开工亿元以上产业项目730个，注册外资实际到账26.7亿美元，引进超亿元市外民资项目130个。围绕推进产业创新转型，着力打造“两基地”，构建以“3+3+N”为重点的现代产业体系，六大产业产值增长10.5%。建成省级以上智能车间25个，宽带IP城域网出口带宽达3300G，获批“宽带中国”示范城市。新增驰名商标6个，获评全国质量魅力城市。 社会消费品零售总额2633亿元、增长10.7%，60家市级以上服务业集聚区营业收入增长20%，新增省级生产性服务业集聚示范区3家，旅游收入、电子商务交易额分别增长15.2%、27.4%。建成市级以上众创空间33家，新增省级以上研发机构35家，国际专利申请量增长45.3%，南通产业研究院孵化能力加速提升，创源科技园开园。围绕支持实体经济发展，实施稳增长6条政策，市本级兑现财政扶持资金13.5亿元，入选全国工业稳增长和转型升级成效明显市。设立“南通企业家日”和“张謇杯”杰出企业家奖，评选表彰“三名”“三创”人物。探索供给侧结构性改革金融创新试点，设立陆海统筹发展基金和中小企业、江海产业发展等二级基金，建立工业存量资产交易平台、中小企业金融服务中心和基金产业园，在全国首发“一带一路”企业集合债券。

　　（二）推进供给侧结构性改革。制定落实“三去一降一补”实施方案，出台工业用地先租后让、棚改货币化安置、政府性债务管理等配套政策。完成省下达的60万载重吨船舶、80万吨钢铁产能化解任务，压减宝通钢铁167万吨产能，坚决落实整治“地条钢”、查处违法违规钢铁产能等工作部署。加强房地产市场分类监管，全市普通商品住宅去化周期缩短至7.3个月。新增直接融资825亿元、地方政府债券及置换债券528亿元。实施全面“营改增”等7大类18条政策，为企业降低成本105亿元。六大领域87个“补短板”项目完成投资340亿元。陆海统筹发展土地利用规划获省政府批准，完成城市（镇）周边永久基本农田划定。推进农业转移人口市民化，出台户籍制度改革意见。成为国家首批海洋经济创新发展示范城市。对接央企合作取得新进展。“三位一体”国企风险监控机制进一步健全。取消行政审批事项37项、职业资格许可和认定事项291项。全面实行“多证合一、一照一码、证照联办”，企业注册登记时间压缩至3个工作日内。探索建设项目市、区联动集中高效审批，实现一般工业项目从立项到施工许可50个工作日内办结。推行“双随机、一公开”抽查机制。

　　（三）加强城乡统筹和生态建设。以国务院批准《南通市城市总体规划（2011-2020）》为契机，开展新一轮城市总规修编研究，制定城市和交通转型发展意见。突出“一主五片两重点”，提高中心城市首位度，启动中央创新区规划和五山及沿江地区生态修复，推进“三城同创”三年行动计划，加快轨道交通前期工作。城建重点工程完成投资199亿元，长江路西延等60个项目竣工，市区新增绿地600公顷。15个重大交通工程完成投资92亿元。北沿江高铁纳入国家规划。编制农业产业空间布局规划，实施规模农业项目365个。获批省高标准农田建设整体推进试点市，新建高标准农田36.4万亩。获评国家级休闲观光农业星级企业21家，新增省级示范家庭农（渔）场37家。农产品出口额全省第一。基本完成农村土地承包经营权确权登记颁证工作，农村产权流转交易市场实现县级全覆盖。实施新一轮生态文明建设三年行动计划，推进288项大气污染防治重点工程和120个水污染防治项目，PM2.5平均浓度降幅全省第一，空气质量优良天数比上年增加16天、达263天，集中式饮用水水源地水质达标率100%。83个循环经济项目完成投资105亿元，实施畜禽粪便沼气发电项目61个。全面落实中央环保督察整改要求，组织开展38项环境突出问题整治，关闭违法违规企业121家。

　　（四）提高开发开放水平。坚决落实“共抓大保护、不搞大开发”的要求，加大沿江非法码头、化工园区等整治力度。推进沿海港产城融合发展，8个重点区镇新开工亿元以上产业项目153个。启动全市域港口一体化改革试点，通海港区集装箱码头开工建设，通州湾港区总体规划环评通过专家审查，吕四港区10万吨级、洋口港区15万吨级航道工程顺利推进。举办江海国际博览会、台商产业转型升级峰会等活动，新引进3000万美元以上外资项目96个。进出口总额2035亿元、增长3.8%，其中出口1517亿元、增长7%，获批中国服务外包示范城市。新增对外投资项目112个，开通“中亚班列”。推进开发园区转型升级创新发展，南通开发区成为长江经济带转型升级示范区，通州湾、洋口港获批筹建省级开发区，海安保税物流中心（B型）获批。市县电子口岸实现互联互通，如东、启东海关和检验检疫顺利开关开检。

　　（五）切实保障和改善民生。加强新形势下就业创业工作，为5235家企业发放稳岗补贴1.46亿元，新增就业8.59万人。企业退休人员养老金、城乡低保、特困人员供养等标准继续提高，残疾人两项补贴制度全面实施。扎实推进教育现代化示范区建设，基础教育优势进一步巩固，教育开放合作取得新进展，在通高校新增对接产业发展需求专业41个。实施市县公立医院综合改革，探索现代医院管理制度和分级诊疗制度，基层医疗服务能力加速提升，智慧医疗便民服务平台全面推广。着力构建现代公共文化服务体系，基层综合性文化服务中心建设有序推进，《梦·江海》等文化精品走出国门。中华慈善博物馆开馆。建成全国友协青少年足球国际交流基地，南通籍健儿连续7届夺得奥运金牌。《南通市志（1983-2005）》出版发行。妇女、儿童、青少年、老年等事业长足发展。夯实社会治理基层基础，“政社互动”实现镇（街道）全覆盖，新增省级和谐示范城市社区26个、农村社区42个。抓好安全生产标准化提档升级扩面，强化安全生产执法，安全生产事故起数、死亡人数继续“双下降”。启动国家食品安全示范城市创建。提升立体化社会治安防控水平，平安寄递物流、基层社会治理“三所共建”等经验全国推广。民族、宗教以及军民融合、国家安全、对口支援和扶贫协作等工作进一步加强。

　　过去一年，我们认真开展“两学一做”学习教育，制定法治政府建设实施方案，稳步推进地方立法，完善政府法律顾问制度，落实“三重一大”决策制度，细化重大行政决策程序，整合建立全市统一的公共资源交易平台。自觉接受市人大及其常委会的法律监督、工作监督和市政协的民主监督。认真办好市人大代表议案、市政协建议案，完成市人大代表建议、政协提案办理任务。支持“万名代表小康行”深入推进，在全面小康社会建设中很好发挥了参谋助手、监督协助、桥梁纽带、示范带头的“四位一体”作用。全面落实党风廉政建设责任制，坚决惩治和有效预防腐败，切实防止“四风”问题反弹。

　　各位代表，本届政府取得的成绩是省委、省政府和市委正确领导的结果，归功于760万江海儿女的团结拼搏，得益于历届政府打下的坚实基础，离不开市人大、市政协的监督支持。我代表市人民政府，向全市人民，向人大代表、政协委员和老同志，向各民主党派、工商联、无党派人士、各人民团体和各界人士，向驻通人民解放军、武警官兵和公安干警及部省驻通单位，向关心支持南通发展的海内外朋友，致以崇高敬意和衷心感谢！

　　我们也清醒看到，南通仍处于爬坡过坎阶段，发展的“痛点”和“短板”还不少。结构性问题仍然突出，实体经济困难较多，有效需求增长乏力，财政收支平衡压力加大，经济总量占全省比重和人均水平还不高，自主创新能力还不强，有影响力的特色产业和龙头企业还不多。生态安全面临挑战，水、大气、土壤环境质量不容乐观，农村面源污染不容忽视，部分行业污染整治不容松懈。社会建设任重道远，城乡居民持续增收难度较大，公共服务资源配置还不均衡，社会安全稳定的风险和隐患增多。政府自身建设还在路上，职能转变还需加快，作风建设长效机制有待完善，政府公务人员干事创业能力与新形势、新任务的要求还有差距。对此，我们将本着对人民、对事业、对历史高度负责的精神，以久久为功的韧劲，勇于攻坚克难，务实加以解决。

　　二、今后五年的奋斗目标和主要任务

　　未来五年，我们肩负着高水平全面建成小康社会的历史重任。省第十三次党代会和市第十二次党代会部署安排了今后五年的目标任务，新一届市政府将以“撸起袖子加油干”的饱满精神，围绕“两聚一高”抓好推进落实。

　　今后五年政府工作的总体要求是：高举中国特色社会主义伟大旗帜，以马克思列宁主义、毛泽东思想、邓小平理论、“三个代表”重要思想、科学发展观为指导，深入贯彻习近平总书记系列重要讲话精神，按照省、市党代会部署，认真落实“五位一体”总体布局、“四个全面”战略布局和五大新发展理念，坚持稳中求进工作总基调，围绕好上又好、能快则快的要求，深化供给侧结构性改革和陆海统筹发展综合改革，推进转型发展，加快富民进程，努力建设“强富美高”新南通，让全市人民共享高水平全面建成小康社会的成果。

　　今后五年政府工作的奋斗目标是：围绕实现“五个更”，力争地区生产总值达万亿元，一般公共预算收入达千亿元，人均地区生产总值超过全省平均水平，科技进步贡献率65.5%，全社会研发投入占地区生产总值比重2.85%；城乡居民收入分别达6万元和3万元，确保2020年、力争2019年建档立卡低收入人口全部脱贫；空气质量优良天数比例74%以上，全面消除黑臭河道、劣V类地表水；文化产业增加值占地区生产总值比重6%，全民思想道德、科学文化素养和健康素质明显提升；法治建设满意度、公众安全感保持全省领先。围绕建设“经济中心、交通枢纽、创新之都、花园城市”，力争基本建成长三角特色产业科技创新基地、长三角北翼具有国际竞争力的先进制造业和现代物流基地，形成公铁水空管多式联运的现代交通运输体系，初步建成国家创新型城市和人才强市、青年和人才友好型城市，成为国家生态园林城市、国家森林城市，让发展水平更高、群众获得感更强。

　　实现以上目标，必须牢牢把握六个方面：

　　（一）聚力创新转型，引领提质增效。坚持把发展作为解决一切问题的基础和关键、把创新作为引领发展的第一动力，紧紧牵住科技创新这个“牛鼻子”，走差别化、特色化的全面创新路子，打造风生水起的创新生态体系，构建以中央创新区为核心、科技园区为载体、创新型企业为主体的全市域创新网络，做大做强做优以“3+3+N”为重点的现代产业，加快高端化、智能化、绿色化、服务化、品牌化步伐，更多依靠技术进步、创新驱动引领发展，在中高速增长中迈向中高端水平。

　　（二）推动协调融合，促进资源集聚。坚持重融合、创特色，按照建设Ⅱ型大城市的目标，以城市、交通转型带动市域城镇体系和城市功能结构优化，提高城乡协调、江海联动、跨江融合发展水平，构建“外成环、内成网”的便捷交通网络，深度对接、服务上海，积极构建（沪）苏通经济圈，努力建设上海“北大门”，加快融入苏南、融入长三角核心区，增强资源要素集聚、辐射功能，显著提升在长三角和扬子江城市群中的竞争力、影响力。

　　（三）加快绿色发展，建设花园南通。坚持“绿水青山就是金山银山”，全面实施“两减六治三提升”专项行动，推动生产生活方式绿色化转型，认真解决群众身边的环境突出问题，有效提供生态公共产品，整体保护山水林田湖生命共同体，使污染排放持续下降、资源节约集约利用水平持续提高、生态环境质量持续好转，让南通天更蓝、地更绿、土更净、水更清、空气更清新。

　　（四）推进开放提升，增创发展优势。坚持双向开放，更加主动融入“一带一路”等国家战略，推进“引进来”与“走出去”深层互动，深入实施开发园区转型升级创新发展战略，积极复制推广自贸区改革经验，构建“六外”协调并进、载体平台国际竞争力显著提升、一类口岸与综保区联动发展的开放体系。

　　（五）聚焦富民惠民，共建美好生活。坚持以人民为中心的发展思想，把增进人民福祉、促进人的全面发展作为一切工作的出发点和落脚点，实施新一轮民生幸福工程和脱贫致富奔小康工程，着力解决群众最关心最直接最现实的利益问题，在充实居民“口袋”、丰富公共服务、维护公共安全、提升文明程度上补短板，巩固教育之乡、文博之乡、体育之乡、长寿之乡、平安之乡等品牌，守牢社会保障、社会救助、公平正义等底线，努力在共建共享中增强市民获得感，在协调均衡中开拓发展新空间。

　　（六）持续深化改革，激发内生动能。坚持把改革作为推动发展的深刺激、强刺激，抢抓国家“支持东部地区在体制机制、陆海统筹等方面率先突破”的机遇，以供给侧结构性改革为主线，提高全要素生产率，扎实抓好土地、海域、港口等资源配置和财税、金融、科技、投融资、国资国企等体制改革，更加注重用行政权力减法换取市场活力加法，统筹经济社会发展各领域重大改革，努力形成系统完备、科学规范、运行有效的体制机制。

　　各位代表，南通正处在转型的“关口”、发展的“风口”。我们将不忘初心、牢记使命、创新实干，紧紧依靠全市人民创造“两聚一高”生动实践，奋力建设充满活力和魅力的新南通！

　　三、2017年主要工作

　　2017年是党的十九大召开之年，是供给侧结构性改革的深化之年，也是全面落实省、市党代会精神的开局之年。我们要抢抓多重国家战略深入实施的新机遇，按照省委对南通建设上海“北大门”的新定位，抬高发展标杆，营造竞争优势，牢牢把握稳中求进工作总基调，深入贯彻省委省政府决策部署和市委要求，奋力改革创新，提升质量效益，确保经济平稳健康发展，在好的前提下力争快一些；确保社会和谐稳定，在稳的基础上力求好一些。

　　今年经济社会发展主要预期目标建议为：地区生产总值增长8.5%左右，全社会研发投入占地区生产总值比重2.65%，高新技术产业产值占规模以上工业比重46.5%，一般公共预算收入同口径增长6%左右，固定资产投资增长10%左右，社会消费品零售总额增长10%左右，进出口总额保持增长，居民消费价格涨幅3%左右，城镇新增就业8万人、登记失业率控制在3%以内，城乡居民收入增长与经济增长同步，节能减排完成省下达目标任务。重点做好八个方面工作：

　　（一）着力保持经济平稳较快增长。把增强企业竞争力和活力作为关键环节，扩大需求、优化供给，夯实稳增长基础。

　　发挥有效投入关键作用。推动31个省级重大项目和市级“双百工程”重大项目早落地、早见效。积极扩大产业投资，新开工亿元以上产业项目500个以上，新增规模以上企业800家。加大基础设施、高新技术、生态环境等投入力度，力争59个交通项目完成投资94亿元，新兴产业、工业技改投资均增长10%左右。落实国家促进民间投资“26条”，积极推动政府和社会资本合作，进一步激发社会投资活力。

　　挖掘消费和外贸增长潜力。大力发展旅游、文化、体育、健康、养老、教育培训等幸福产业和服务经济，打造新的消费热点。开展增品种、提品质、创品牌专项行动，优化消费供给，培育全国知名品牌示范区，新增驰名著名商标33个、省名牌产品75个。推进“互联网+商贸”，丰富商业业态，方便和引导群众消费。落实外贸稳增长、调结构政策措施，支持企业开拓新市场、提升竞争力。建好综保区、开放口岸、出口基地、进口交易中心等平台，壮大市场采购贸易方式、跨境电商、外贸综合服务、飞机保税维修等新业态，扩大服务贸易，促进外贸优进优出。拓展电子口岸功能，加快国际贸易“单一窗口”建设，提高贸易便利化水平。

　　倾力服务实体经济发展。构建“亲、清”新型政商关系，以“店小二”精神对重点产业和企业实施精准帮扶。提高政银企合作成效，有效增加制造业和小微企业贷款，完善企业应急转贷机制，创新发挥工业存量资产交易、中小企业金融服务等平台作用。鼓励和支持企业借助多层次资本市场做大做强，新增上市公司、“新三板”挂牌企业20家以上。丰富“南通企业家日”活动内容和方式，继续组织好“张謇杯”杰出企业家和“三名”“三创”人物、市长质量奖评选，引导企业家专注创新创业，发扬“工匠精神”、打造“单项冠军”、培育“百年老店”。

　　（二）着力加快产业转型升级。坚持扩增量与优存量并举，加快发展新经济，促进新业态新模式与传统优势产业融合发展。

　　做强做大“3+3”重点产业。整合产业资源，集聚高端要素，打造有世界影响的重大平台和展会品牌，培育具有核心竞争力的企业集群，力争规模以上工业总产值增长8.5%，“3+3”产业产值超过1万亿元。推动船舶海工向高端绿色、关键配套方向发展，采用新标准的船舶比重达60%。加快高端纺织向智能型设计、定制化生产、个性化服务转型，启动通州湾高端纺织产业园建设。加强新技术研发，提升电子信息发展水平，力争产值超过2300亿元。实施智能制造行动，促进智能装备发展，培育工业机器人、3D打印等新产业，新增市级以上智能车间20个。聚焦先进高分子、特种金属材料等细分领域，布局石墨烯、碳纤维等前沿产业，促进新材料产业集聚发展。推进新能源及新能源汽车“三园三区”建设，加快形成从动力电池到整车分工协作的产业链。

　　做精做专特色产业。加快建筑产业现代化，发展装配式绿色智慧建筑，培育全产业链、国际化企业，让建筑铁军“智勇双全”。突出全域旅游战略，实施旅游业发展三年行动方案，推进19个重大旅游项目和100个乡村游项目。积极培育通用航空产业，推动低空通航装备研发应用。传承发展红木雕刻、祖传医药等南通元素历史经典产业。推进“互联网+”，发展大数据、云计算等新产业新模式，在数字经济、创意经济、分享经济、新金融等领域求突破，宽带IP城域网出口带宽达4000G。规划建设一批有明确产业定位、文化内涵、旅游功能、社区特征的特色小镇。

　　做优做特现代服务业。突出项目投入、企业培育，壮大现代物流、金融服务、休闲旅游、电子商务、工业设计、文化创意等重点产业，服务业增加值占地区生产总值比重达48%以上。推进服务业集聚区提档升级，力争60家市级以上集聚区实现营业收入3900亿元。大力发展总部经济、平台经济、楼宇经济，确保平台经济示范企业达30家，市区纳税超亿元楼宇达20幢。加快建设中国服务外包示范城市，服务外包执行额增长30%以上。

　　（三）着力构建创新生态系统。以便利、宽松、包容为导向，提升创新浓度，厚植创新土壤，加快培育创新型增长点。

　　强化产业科技创新。围绕产业转型升级部署创新链，新增省级以上企业研发机构25家。搭建开放共享的科技公共服务平台，建设检验检测产业园，打造技术转移和交易、产业中试、共性技术研发等平台。开展50项重大产业技术集中攻关，承接一批国家科技重大专项和省重大科技成果转化项目。支持企业与大院大所、知名高校开展合作，实施产学研协同创新项目500项。

　　壮大创新创业主体。推进创新型企业梯队培育行动，高新技术企业突破1000家。支持企业加大研发投入，规模以上工业企业研发经费支出增长12%。落实“人才8条”政策，提升江海英才创业周、国际创业大赛等引智聚才作用，新增国家“千人计划”人才20名、省“双创”人才30名。推进高技能人才“十百千万”培育工程。实施“通籍英才归雁计划”，引导南通籍人才、企业家、高校毕业生返乡创新创业，办好留学人员创业园，壮大创新创业“新四军”队伍，培育本土优秀“创客”。

　　优化区域创新布局。打造高水平创新平台，抓好中央创新区“四中心一板块”规划建设，启动科创中心一期工程。推动高新区创新发展、争先进位，加快形成“一区一战略产业”布局。发挥南通产业研究院、创源科技园引领作用，形成互联网、智能装备等产业集聚效应。建设众创社区，增强众创空间集成创新和产业链配套能力，新增省级以上众创空间5家。

　　深化科技体制改革。落实省“科技创新40条”政策措施，完善科技成果转移转化机制，建立线上线下融合的技术市场。发挥陆海统筹发展基金引导作用，构建“政府引导基金+母基金+子基金”运作模式，加快科创基金市场化、专业化，撬动更多资本投向新经济、新产业。推进国家知识产权投融资试点工作，构建知识产权综合管理体制，力争万人发明专利拥有量达20件。

　　（四）着力推进新型城镇化和城乡一体化。加大城市工作力度，提升城镇综合承载和服务能力，促进城乡统筹协调发展。

　　提升中心城市能级。实施新一轮城市总体规划修编，推进“多规合一”，加快城市功能核和景观核建设，增强对人口、人气的集聚能力。落实“三城同创”年度任务。抓好增绿、清水、畅通、便民四大行动，实施总投资1000亿元以上、年度投资300亿元左右的城建重点工程。加强公园绿地建设，实施38个“空转绿”工程。开展新一轮水环境综合整治三年行动。加快建设机场大道、啬园路等快速路，实施跃龙路拓宽改造等26项骨干路项目和一批支路项目，新增道路里程17公里。扎实做好轨道交通1号线建设前期工作，确保一期工程年内开工。完善公共服务设施规划，健全社区服务体系，缓解“停车难”等问题。加强重点区域规划建设。重视老城区改造提升，推动寺街、西南营和唐闸等历史文化街区保护与修缮。统筹中央创新区建设和中央商务区提升。推进五山及沿江地区生态修复，力争五山通过国家5A级景区创建资源评审。加快南通火车西站枢纽片区功能建设。提升星岛水岸、先锋生态涵养片区绿化景观。增强管理与服务能力。实施23项公用事业项目。依法加强濠河景区管理。推进地下综合管廊试点、海绵城市示范项目和湿地系统建设。大力发展公共交通。打好街景提升、拆违控违等攻坚战，开展背街后巷、农贸市场和城郊结合部等环境专项治理，强化小区物业管理。

　　推进农业供给侧结构性改革。以高标准农田建设整体推进试点为抓手，推动农业规模化、产业化、现代化。深入实施国家现代农业示范区“863”工程，力争规模农业项目投入超过150亿元，新增高效设施农（渔）业7.5万亩。提升“菜篮子”保供能力，确保农产品质量安全。积极推动农副产品进上海。大力培育新型农业经营主体，新增市级以上示范家庭农（渔）场30家。推进粮食生产全程机械化。推广“一村一品一店”，加快培育电商平台、示范主体，农村电子商务销售额增长20%。

　　创新城乡统筹体制机制。加快实施6项新型城镇化综合试点，在统筹城乡产业发展、基础设施、要素配置、公共服务、就业社保等方面争取新突破。推进省级农村土地“三权分置”示范县创建，深化农村集体产权制度改革。支持各县（市）城增强支撑和带动能力。开展市级中心镇、沿海“区镇合一”镇特色打造与环境改善行动。加强农村公路、桥梁等建设改造，深化农村环境“四位一体”长效管理，创建11个省级美丽乡村、三星级康居示范村。

　　（五）着力推动跨江融合、特色发展。把靠江靠海靠上海的优势进一步转化为发展优势、富民优势。

　　加快对接上海、区域融合。围绕行政推动常态化、区域市场一体化、社会服务同城化、交通运输便利化，强化产业合作和园区共建。在沪举办对接服务上海大会，承接高端制造、金融和服务外包等“溢出”效应，加强农业、旅游、建筑业等服务配套，力争引进亿元以上先进制造业、服务业项目各100个。全方位对接上海创新资源，加快“上海孵化、南通转化”。推动市北高新等7个沪通合作园区提升功能，重点推进通州湾示范区、南通综保区与上海对接，力争实现各县（市）区沪通合作园区全覆盖。策应“崇明世界级生态岛”建设，启动东平-海永-启隆城镇圈协同规划编制，加强与上海的生态保护和生态经济合作。加快苏通、锡通等跨江合作园区体制机制创新，探索利益共享新模式，导入更多优质资源和项目。做好与新疆伊宁、青海贵德等对口支援和与陕西汉中的扶贫协作工作。

　　深化陆海统筹、江海联动。落实长江经济带发展规划对南通的要求，推进沿江共抓大保护“一张图”和产业改造提升计划，实施沿海“风光带建设十百千万工程”“特色产业培育123计划”和“美丽小镇打造123行动”。按照“一市一港一集团”的思路，整合沿江沿海港口资源，推动管理、经营、集疏运一体化，加快通海、如皋港区和沿海三大港区航道、泊位建设。推动通州湾示范区在江海联动合作、陆海统筹改革、海洋经济发展上先行先试，强化港口、产业、城市一体化发展。推进海洋经济创新发展示范城市建设，壮大装备制造、海洋生物、现代物流、滨海旅游等重点产业，海洋生产总值增长9.5%。

　　强化综合交通支撑和带动。完善综合交通运输体系发展规划，推动各种运输方式互联互通。加快沪通铁路及沪通长江大桥、宁启铁路二期建设，力争开工盐通铁路、平东铁路货场项目，积极推动通苏嘉城际、北沿江高铁前期工作。推进海启高速、锡通高速北接线及宁通高速扩容等项目。实施机场航站区改扩建工程。积极布局江海河联运项目，加快通扬线等高等级航道建设。

　　（六）着力深化改革开放。“挑最重的担子、啃最硬的骨头”，狠抓各项重点举措落实，进一步增强发展动力和活力。

　　深入推进供给侧结构性改革。优化存量、引导增量、主动减量，推动“三去一降一补”取得更大进展。运用市场、标准、法治等手段，有效化解过剩产能，推进船舶行业结构调整、产能合作；深入实施淘汰落后产能、违法违规项目清理和联合执法三项行动，严禁违规新增钢铁产能，保持对“地条钢”等非法生产经营行为打击的高压态势，分别压减印染、钢丝绳落后产能5000万米、15万吨。制定化工产业转型发展意见，淘汰化工落后产能，推动沿江绿色化工企业向沿海园区转移、重点区域化工企业关停并转迁，确保化工生产企业入园率54%。坚持房子的居住属性，稳房价与去库存并重，优化供应结构，加大监管力度，保持房地产市场平稳健康发展。支持企业去杠杆、债转股，新增直接融资300亿元，严密防范不良资产、政府债务、企业互保联保等风险，有序推动政府融资平台市场化转型。挖掘企业税费、生产要素、制度性交易等降成本空间，减轻实体经济负担110亿元。加快推进六大领域87个“补短板”项目，完成投资350亿元。

　　推动重点领域改革落地见效。抓好陆海统筹发展综合改革试点，统筹各项国家级、省级改革试点。调整完善土地利用总体规划，统筹配置建设用地与用海，推进低效用地再开发和村庄建设用地布局调整。推动国家海域综合创新示范市建设，深化开发性金融促进海洋经济发展试点。加大财税体制改革力度，提高财政资金资本化运作水平。加快金融创新试点，争取设立地方法人保险机构，在投贷联动、产业基金平台建设、金融支持企业“走出去”等方面加快突破。深入推进国资国企改革，优化国有资本投向，加强与央企对接。健全现代市场体系，深化价格改革。

　　拓展对内对外开放空间。对接“一带一路”沿线重点国家和地区，鼓励船舶海工、家纺、建筑等优势企业“走出去”，开展跨国并购和产能、装备制造合作，支持境外经贸合作园区建设。坚持引资、引技和引智结合，强化产业链、专业化招商，办好江海国际博览会、世界通商大会、台商转型升级峰会等品牌活动，力争注册外资实际到账25亿美元，引进超亿元市外民资项目140个。推进开发园区转型升级创新发展“六大工程”，推动国家级、省级园区集聚高端产业、平台和要素，支持现有园区升格升级。

　　（七）着力改善生态环境质量。扎实推进“两减六治三提升”专项行动，让广大群众感受到环境提升的实际成效。

　　严格生态空间管控。以主体功能区规划为基础，严守农业和生态空间保护红线。建立生态环境损害赔偿制度，实行全流域水环境区域补偿。落实污染物排放许可制度，推进碳排放权交易以及排污权有偿使用和交易。扩大化工产业负面清单范围，探索区域环评与项目环评联动机制。持续开展环保执法“亮剑行动”，对各类环境违法行为零容忍、严打击。

　　从源头削减污染排放。减少煤炭消费总量，压减燃煤发电和热电机组以及非电用煤，发展清洁能源，提高燃煤锅炉整治标准。落实国家循环发展引领计划，推进总投资550亿元的83个循环经济重大项目，全面推动省级以上开发园区创建生态园区。鼓励节能环保技术、装备和服务产业化，实施100个重点减排项目和100个节能技改项目。推广绿色建筑、新能源汽车等环保产品，深化各类绿色创建，倡导绿色低碳生活。

　　协同治理环境突出问题。主要是“六治”：治区域水环境，全面落实河长制，实行最严格的水资源管理制度，加强重点区域、重点行业、重点断面治理；治垃圾，开展“六个一”行动，加快垃圾源头分类和19个处置工程建设；治黑臭水体，控源截污、内源治理、生态修复、长效管护多管齐下，促进水质明显改善；治畜禽养殖污染，依法关停禁养区养殖场，规模养殖场治理率达65%；治挥发性有机物，完成重点化工园区和企业废气排放源整治，控制机动车船、建筑扬尘等污染；治环境隐患，限期清理饮用水水源地内违法违规项目，推进6个危废处置工程，制定土壤污染防治方案。抓好中央环保督察反馈问题长效整改，推进33个环境突出问题整治。

　　加强生态修复与保护。坚守长江沿岸生态等“三条红线”、流域布局等“六条底线”，落实近岸海域污染防治方案，推进海洋生态文明示范区建设重点项目，省控以上地表水断面达标率64.5%以上。落实生态红线区域保护措施，实施60个重大生态保护和修复工程，编制生物多样性保护规划，完成生物多样性物种普查。构建“六廊五圈多点”生态安全屏障，推进国家森林城市建设重点工程，新增造林面积10万亩，自然湿地保护率达45%。

　　（八）着力提升民生获得感。从事关群众切身利益的突出问题入手，进一步加大投入力度，办好11件50项重点民生实事，力争全市财政民生支出占比提高1个百分点。

　　拓宽居民增收渠道。落实聚焦富民各项政策措施，激发重点群体增收活力，千方百计提高城乡居民工资性收入和经营、财产、转移净收入。推动大众创业、万众创新，实施万人创业计划、大学生就业促进和创业引领计划，加强省级创业型乡镇（街道）建设，办好人力资源服务产业园。建立城乡一体的就业援助机制，强化就业技能培训，动态消除城镇零就业和农村零转移贫困家庭。抓好精准扶贫、精准脱贫，推进92个经济薄弱村扶贫项目，加强老区建设，年收入7000元以下建档立卡低收入人口脱贫率达55%以上，其中6000元以下低收入人口全部脱贫。

　　加大社会保障力度。扩大全民登记参保覆盖面。稳步提高企业退休人员和城乡居民基本养老保险待遇，深化机关事业单位养老保险制度改革。落实医疗保险城乡统筹措施，完善医疗保险政策。巩固市区基本照护保险实施成果，推动县级试点。继续实施多元安置，扎实推进市区棚户区改造三年计划。完善社会救助体系，有效帮助贫困人口和有困难的群众解决实际问题。

　　加强文化强市建设。把践行社会主义核心价值观与弘扬新时期南通精神紧密结合，繁荣江海特色文化，创新发展哲学社会科学。深化群众性精神文明创建，加强文明城市长效管理，提升城市文明程度和市民综合素质。实施40个文化产业重大项目，做强重点文化产业集聚区和骨干文化企业，文化产业增加值占地区生产总值比重超过5%。推进文化惠民，创建省级公共文化服务体系示范区，开工建设南通大剧院、美术馆等重大文化设施，加快基层综合性文化服务中心建设；倡导全民阅读，加强科普工作，建设“书香南通”；举办市第十一届运动会暨第二届全民健身运动会，推动校园足球、社会足球发展。

　　协调发展社会事业。优先发展教育，加强学前教育，改善义务教育学校大班额现象，加快普通高中优质特色发展，提升职业教育服务产业发展能力；推进高等教育国际合作办学，支持南通大学建设有特色高水平教学研究型大学；完善终身教育体系，发展特殊教育、老年教育、民办教育，提高教育信息化水平。建设健康南通，深化公立医院综合改革，推进分级诊疗，建成各县（市）医疗集团和5个市级专科医疗联盟，家庭医生签约服务覆盖率30%以上；探索基层卫生人才县乡村一体化管理，新增全科医生120名；实施健康医疗大数据工程，完善计划生育管理与服务。强化养老服务，抓好医养结合试点，提高社区居家养老服务水平，每千名老人养老床位数达35张。促进妇女、儿童、青少年、关心下一代和老年等事业发展，加强慈善、红十字、残疾人等工作。

　　提高社会治理水平。强化公共安全意识，健全安全生产责任体系、安全风险分级管控和隐患排查治理体系，落实食品药品安全监管“四个最严”，有效防范公共安全事件和事故。严格社会治安综合治理领导责任制、法治建设工作领导责任制和信访工作责任制，推动技防体系提档升级，发挥市综治中心和社会治理大数据中心作用，加强重点区域、重点人群精准服务管理。强化源头治理，引导群团组织、社会组织、社区组织和基层群众开展民主协商，完善社会矛盾纠纷多元化解机制，加强诚信南通、和谐社区建设。落实法治建设重要举措实施规划，深入开展“七五”普法，争创全国法治城市创建先进市。推动军民深度融合发展，强化国家安全和双拥、民族、宗教、档案、保密等工作。

　　四、加强政府自身改革建设

　　扎实推进政府治理体系和治理能力现代化，加快建设服务型政府、法治政府和廉洁政府，保障各项目标任务有效落实。

　　（一）坚持为民施政，加快转变职能。以群众和企业需求为导向，健全“集中高效审批、分类监管服务、综合行政执法”的政府治理架构。深化简政放权，把市场能有效调节的交给市场，企业能自主决策的还给企业，基层服务管理更方便有效的放给基层。落实“减证便民”专项行动，进一步减事项、减流程、减收费。加快审批标准化、扁平化、便利化，优化证照联办、联合审图、多评合一等流程，健全预审查、帮办代办等服务机制，推进两级审批服务平台互联互通和审批联动。完善综合有效的监管体系，分类推进综合行政执法，实现“双随机、一公开”抽查全覆盖。加强政务服务体系建设，推进政府信息资源整合和数据共享，实施“互联网+政务服务”135行动，力争网上办件量超过60%，让群众少跑腿、好办事。牢固树立公仆意识，身体力行富民强企奔小康“走帮服”和“万名代表小康行”。

　　（二）强化依法行政，自觉接受监督。健全依法行政制度体系，提高政府立法质量。优化依法决策机制，完善重大行政决策事项清单，加强合法性审查。建立健全政务公开、信息发布和舆论引导制度，及时回应社会关切。严格实施权力清单、责任清单动态管理，全面落实行政执法责任制。依法接受党内监督和人大及其常委会的监督，主动接受政协的民主监督，认真办好人大代表议案、建议和政协建议案、提案。推进公共资金、国有资产、国有资源和领导干部经济责任履行情况审计全覆盖，重视社会监督和舆论监督，以全方位监督促进透明运行。

　　（三）倡导高效勤政，务实担当作为。完善政府工作“推进、协调、督查”体系，强化“钉钉子”精神，弘扬“马上就办”作风，在抓细落小上见实效。落实改革创新容错纠错机制，为勇于创新者鼓劲，为敢于担当者撑腰。健全督查激励长效机制和行政问责机制，推进重大政策举措第三方评估，对真抓实干、成效明显的加大激励力度，大力倡导敬业奉献；对落实不力的严肃追究责任，坚决整治“为官不为”，以奖惩分明促进勤政有为。

　　（四）保持清廉从政，严守纪律规矩。牢固树立“四个意识”，始终坚持在市委领导下开展工作，严格遵守政治纪律和政治规矩，把全面从严治党要求落实到政府工作的各方面。切实加强政府系统党的建设，从严落实主体责任，全面履行“一岗双责”，以更高标准抓好党风廉政建设。深入落实中央八项规定精神和省、市委十项规定，严控“三公”经费支出，加强党政机关和事业单位办公用房、公务用车管理，坚持不懈纠正“四风”。严格政府“三资”监管和投资管理，推进公共资源交易市县一体化，更好防控廉政风险。筑牢拒腐防变的思想道德防线，保持有腐必惩、有贪必肃，促进干部清正、政府清廉、政治清明。

　　各位代表，发展没有坦途，奋斗成就精彩。让我们更加紧密地团结在以习近平同志为核心的党中央周围，在省委、省政府和市委的坚强领导下，开拓创新，励精图治，为高水平全面建成小康社会努力奋斗，以优异成绩迎接党的十九大胜利召开！
